# Brain network topology and personality traits: A source level magnetoencephalographic study

**DOI:** 10.1111/sjop.12835

**Published:** 2022-06-08

**Authors:** Emahnuel Troisi Lopez, Valentina Colonnello, Marianna Liparoti, Mauro Castaldi, Fabio Alivernini, Paolo Maria Russo, Giuseppe Sorrentino, Fabio Lucidi, Laura Mandolesi, Pierpaolo Sorrentino

**Affiliations:** ^1^ Department of Motor Sciences and Wellness University of Naples “Parthenope” Naples Italy; ^2^ Department of Experimental, Diagnostic and Specialty Medicine, Alma Mater Studiorum University of Bologna, Policlinico S. Orsola‐Malpighi Bologna Italy; ^3^ Department of Social and Developmental Psychology, Faculty of Medicine and Psychology University of Roma “Sapienza” Rome Italy; ^4^ Institute for Diagnosis and Cure Hermitage Capodimonte Naples Italy; ^5^ Department of Humanities University of Naples “Federico II” Naples Italy; ^6^ Institut de Neuroscience des Systemès, Aix‐Marseille University Marseille France

**Keywords:** Brain network, Cloninger, functional connectivity, magnetoencephalography, temperament and character inventory

## Abstract

Personality neuroscience is focusing on the correlation between individual differences and the efficiency of large‐scale networks from the perspective of the brain as an interconnected network. A suitable technique to explore this relationship is the magnetoencephalography (MEG), but not many MEG studies are aimed at investigating topological properties correlated to personality traits. By using MEG, the present study aims to evaluate how individual differences described in Cloninger's psychobiological model are correlated with specific cerebral structures. Fifty healthy individuals (20 males, 30 females, mean age: 27.4 ± 4.8 years) underwent Temperament and Character Inventory examination and MEG recording during a resting state condition. High harm avoidance scores were associated with a reduced centrality of the left caudate nucleus and this negative correlation was maintained in females when we analyzed gender differences. Our data suggest that the caudate nucleus plays a key role in adaptive behavior and could be a critical node in insular salience network. The clear difference between males and females allows us to suggest that topological organization correlated to personality is highly dependent on gender. Our findings provide new insights to evaluate the mutual influences of topological and functional connectivity in neural communication efficiency and disruption as biomarkers of psychopathological traits.

## INTRODUCTION

Personality can be defined as “the sum of all the characteristics that make a person unique” (Weinberg & Gould, 2015). This uniqueness depends on the interaction of genetic and environmental factors, and it is not surprising that every model and taxonomy that has attempted to describe the diversity of personality characteristics has considered this interrelationship (Weiner, Tennen, & Suls, 2013). One such model that has made a significant contribution is Cloninger's psychobiological model, which identifies four primary‐basic personality temperaments (Novelty Seeking, NS; Harm Avoidance, HA; Reward Dependence, RD; and Persistence, P) and three characters (Self‐Directedness, SD; Cooperativeness, CO; and Self‐Transcendence, ST) that are all measurable with the Temperament and Character Inventory (TCI) (Cloninger, Svrakic & Przybeck, 1993). While the temperaments in Cloninger's model are generally associated with genetic substrates, the characters concern individual aspects linked to learning and socio‐cultural factors. It has been suggested that psychophysical well‐being depends on the harmonious development of these traits and characters (Cloninger & Zohar, 2011). In this framework, Cloninger's psychobiological model represents key descriptors of human behavior. In fact, the “direction of the action *toward*” can be identified in NS, the “direction of the action *away from*” in HA and the “maintaining of the behavior” in RD and P (Laricchiuta Petrosini, Piras *et al*., 2014). The TCI is therefore a sensitive instrument for evaluating nonadaptive behaviors, as the occurrence of an excessive tendency toward a specific temperamental trait could indicate psychopathological disorders (Abram & DeYoung, 2017). As a supportive example, anxiety disorders and depression are often correlated with high HA scores (Biederman, Hirshfeld‐Becker, Rosenbaum *et al*., 2001; Muris, Merckelbach, Schmidt, Gadet & Bogie, 2001), high NS and HA scores are very frequent in individuals with dependences and schizophrenia (Sim, Kim, Yim, Cho & Kim, 2012), and high HA and low NS scores are associated with somatization disorders (Karvonen, Veijola, Kantojärvi *et al*., 2006).

The last decade has seen a growing interest in studying the biological substrates of individual differences in order to explore how the diversified variety of human behaviors arise from different neural patterns and how they are correlated to specific cerebral structures or neurotransmitter systems (Booth, Mõttus, Corley *et al*., 2014; DeYoung, Hirsh, Shane, Papademetris, Rajeevan & Gray, 2010; Riccelli, Toschi, Nigro, Terracciano & Passamonti, 2017). Precisely, for this new approach to the study of personality, it is increasingly the common terminology of “personality neuroscience” to derive explanatory models of individual differences that are based on the idea that an individual cannot be understood without understanding his/her structural and functional brain characteristics and fluctuations in neurotransmitter levels (DeYoung *et al*., 2010; DeYoung & Gray, 2009; Markett, Montag & Reuter, 2018). For example, the tendency toward exploratory activity and the intense excitement for new stimuli identified in NS is associated with low dopaminergic activity, while the response to avoid punishment and non‐reward identified in HA is correlated with high serotonergic activity (Mincic, 2015). The relationship between individual differences and variability in brain function and structure has yielded results that are not always consistent. Added to that is the variability in the samples and the diversity of the questionnaires used to investigate personality traits. However, neuroanatomical and neurofunctional studies have often evidenced the involvement of cortico‐limbic pathways and basal ganglia in relation to specific personality traits (Deckersbach, Dougherty & Rauch, 2006; Gogtay, Sporn, Clasen *et al*., 2004; Kumari, Ffytche, Williams & Gray, 2004). Recently, a cerebellar involvement has been observed in sustaining motivational temperamental traits. In fact, it correlates positively with NS scores and negatively with HA scores (Laricchiuta *et al*., 2014; Petrosini, Cutuli, Picerni & Laricchiuta, 2017; Picerni, Petrosini, Piras *et al*., 2013).

Newly, the personality neuroscience has specialized in investigating the correlation between specific behavioral styles and the efficiency of large‐scale networks during a resting state condition from the perspective of the brain as a coherent interconnected network (Sporns, Tononi & Kötter, 2005). Indeed, analyzing personality traits during resting state is possible because they are stable over longer stretches of an individual's lifespan (Edmonds, Jackson, Fayard, & Roberts, 2008; Specht, Egloff & Schmukle, 2011). Although the “resting brain is never truly resting” (Seeley, Menon, Schatzberg *et al*., 2007), in resting participants, it was still possible to identify a large‐scale network critical for emotional salience processing. That network, called the “insular salience network,” is located around the anterior insula including the anterior cingulate, parts of the basal ganglia, and cortical regions along the operculum, and it receives input from the amygdala (DeYoung *et al*., 2010; Markett *et al*., 2018; Seeley *et al*., 2007).

However, most studies in this context take into account other personality models using brain connectivity measures determined through hemodynamic neuroimaging techniques, such as functional MRI (fMRI), and positron emission tomography (PET), and electroencephalography (EEG).

A suitable technique to investigate the link between personality traits and brain regions within the whole brain network is magnetoencephalography (MEG). MEG is a promising functional neuroimaging technique that allows researchers to record brain magnetic activity and to identify patterns of neural oscillations in several bands with highly accurate temporal resolution (Baillet, 2017) compared to fMRI and PET. Furthermore, MEG signals are unaffected by distortion, while in EEG electric currents become distorted as they pass through the skull and other tissues (Leahy, Mosher, Spencer, Huang, & Lewine, 1998). The MEG signal reflects integrated synaptic activity with high fidelity and thus offers a highly accurate measure of brain activity (Hämäläinen, 1992). In addition to estimating the connectivity among brain regions, exploring each region's role within the brain network is vital. Graph theory, a field of mathematics that studies network structures, can be usefully exploited to explore the topological characteristics of the brain (Bullmore & Sporns, 2009).

To date, research on resting‐state neural correlates in relation to Cloninger's personality traits is limited. The present study therefore investigates the brain topology correlated to Cloninger's temperaments and characters in healthy individuals by means of MEG, which allows detection of the complex dynamic properties of brain networks.

To the best of our knowledge, there have been only two MEG studies aimed at characterizing the topological properties and functional connectivity correlated to personality traits. However, neither can be directly compared to our study. One study considered a psychopathological population (James, Engdahl, Leuthold, Krueger & Georgopoulos, 2015), while the other used a different personality model (Kabbara, Paban, Weill, Modolo & Hassan, 2020).

In the present study, we examine how individual differences in temperaments and characters described by Cloninger are correlated with specific cerebral structures in healthy subjects during a resting state condition, considering the brain as a coherent interconnected network. Because this is the first MEG study to analyze topological properties in healthy subjects using Cloninger's model, we did not make specific predictions about trait‐specific network activity, though we did expect an involvement in the cerebral areas of the insular salience network that are correlated to HA, since the personality trait is mainly related to salience stimulus.

Furthermore, given that our sample consisted of both men and women, we expected to find some gender differences in TCI and its relationship with topological characteristics, as it has been demonstrated that gender is a fundamental factor to consider when trying to understand the morphological underpinnings of inter‐individual differences in personality traits (Nostro, Müller, Reid & Eickhoff, 2017).

## METHODS

### Participants

Fifty healthy participants were enrolled (20 males, 30 females, mean age: 27.4 ± 4.8 years). Exclusion criteria were age > 40 years, left‐handed, personal history of neurologic, psychiatric or psychological disease, and the consumption of psychoactive drugs. All participants provided written informed consent. The study was carried out in accordance with the Declaration of Helsinki and was approved by the Local Ethics Committee of the University of Naples “Federico II” (Prot. n. 17/2021).

### Temperament and character inventory (TCI)

Participants completed the Temperament and Character Inventory (TCI) consisting of 240 items true/false questionnaire (Cloninger *et al*., 1993). TCI is a self‐rated instrument that provides a comprehensive inventory of dimensions of temperament (Novelty Seeking, NS; Harm Avoidance, HA; Reward Dependence, RD; Persistence, P) and character (Self‐directedness, SD; Cooperativeness, C; Self‐transcendence, ST). NS, HA, and RD consist of four sub‐scales, while P has only one. In general, NS refers to the tendency to seek novel stimuli and experiences; HA refers to a tendency to inhibition of behavior in response to signals of punishment; RD refers to a tendency to the maintenance of behavior in response to cues of social reward, and P measures the tendency towards perseverance in the face of adversity. For that concerns the three domains of character, SD measures the ability to use the willpower necessary to achieve personal goals; C measures the ability to cooperate with others; ST measures the ability to look beyond self‐interest to see oneself as part of a larger whole, which could be described as a marker of maturity but could also be described as a tendency towards spirituality or a belief in the religious or supernatural.

The items are divided into positive and negative items and the scoring for each item is dichotomous (TRUE/FALSE), with a 1/0 score for positive items and 0/1 for negative items. The sum of the points obtained gives a “Raw Score” to which it is possible to match a “Percentile Score” and a “T‐Score” which, shown on the graph, allows defining a personality profile. A percentile value above 67% and one below 33% are, according to the literature, considered abnormal (Cloninger *et al*., 1993).

Whenever an answer (true or false) was in accordance with a specific trait, a point was assigned. The final score was calculated as the ratio between the total score and the total number of items composing a specific trait.

### Acquisition

**Fig. 1:**
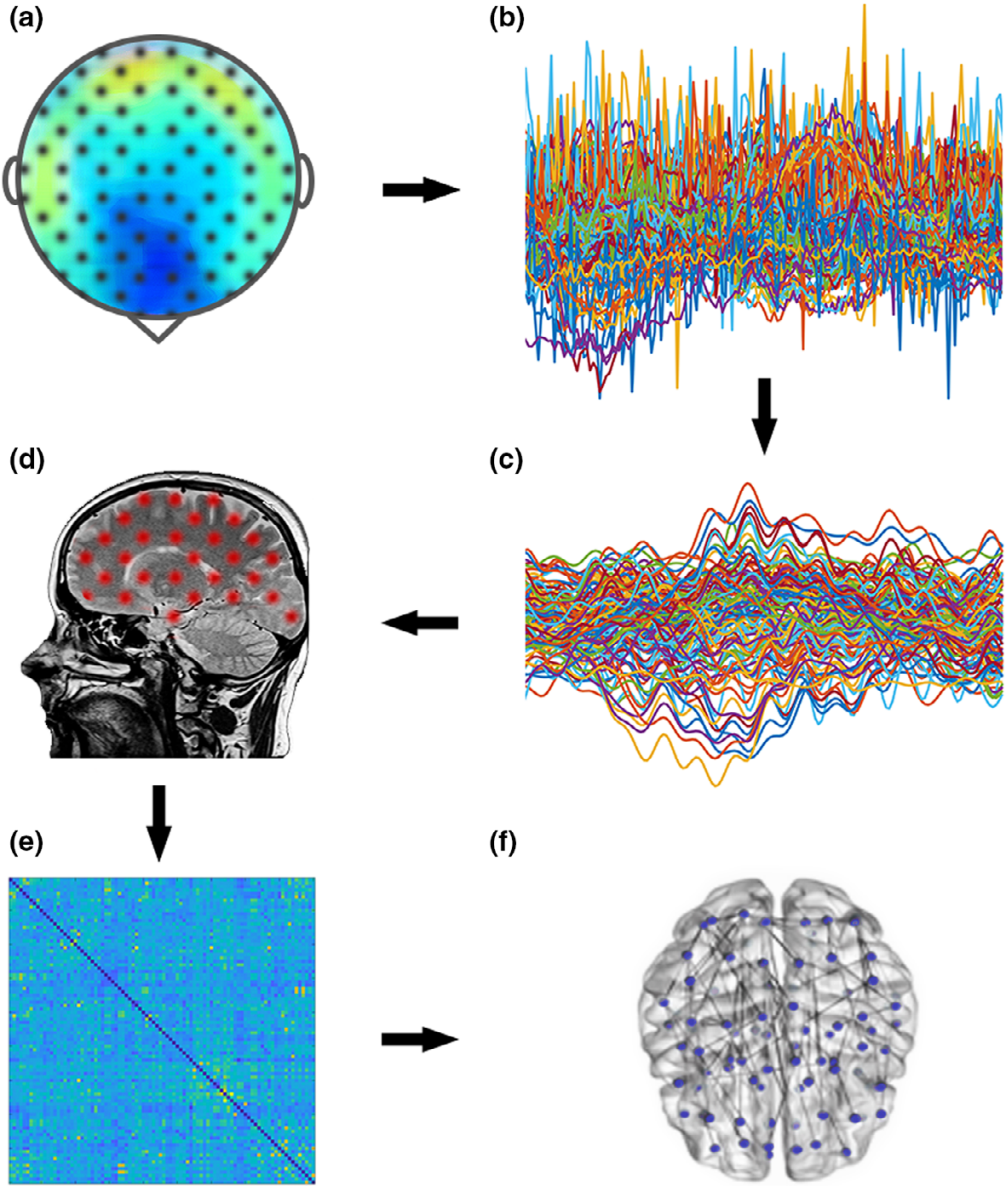
Schematic illustration of the reconstruction of MEG signals.a. Brain activity recording through magnetoencephalography (MEG). b. Noisy signals identification. c. Cleaned signals. d. Structural MRI and MEG signals co‐registration. e. Connectivity matrix calculated using Phase Linearity Measurement. f. Brain network obtained through Minimum Spanning Tree. [Colour figure can be viewed at wileyonlinelibrary.com]

Participants were examined with a MEG system composed of 163 magnetometers. The system was developed by the National Research Council at the Institute of Applied Sciences and Intelligent Systems “E. Caianiello,” Pozzuoli, Naples. For each participant, four coil positions (two on the forehead, two behind the ears) and four anatomical references (nasion, right and left preauricular points, vertex) were digitally recorded prior to acquisition using Fastrak (Polhemus®) (Sorrentino, Seguin, Rucco *et al*., 2021b). The coils were activated and localized at the beginning of each registration segment. The participant was seated in a magnetically shielded room, and brain activity was recorded in a resting state, with eyes closed, in two separate segments of 3′35″ each. To detect and remove artifacts, cardiac and ocular activity was recorded during MEG acquisition (Gross, Baillet, Barnes *et al*., 2013). Sample frequency was 1,024 Hz, and signals were filtered through a smoothing filter in the 0.5–48 Hz band (Fig. 1a).

### Preprocessing

To reduce environmental noise, we used the principal component analysis (Sadasivan & Dutt, 1996) available within the FieldTrip toolbox (Oostenveld, Fries, Maris & Schoffelen, 2011); the noisy channels and segments were removed by an experienced operator after visual inspection. Subsequently, cardiac and ocular activity was removed as an artifact through independent component analysis (ICA), performed for each subject individually (Barbati, Porcaro, Zappasodi, Rossini & Tecchio, 2004). Specifically, the signals were projected on a new set of bases with the constraint of making the components maximally independent. This was done using the fast‐ICA algorithm as implemented in the FieldTrip toolbox (Oostenveld *et al*., 2011). Once the signals were decomposed, a visual inspection of the component time‐courses and the spatial topography allowed the recognition of physiological artefacts. In simple terms, the shape of the components (and their timing) was visually compared to the recorded electrocardiogram and electrooculogram signals, similarly previous MEG studies (Rucco *et al*., 2021; Sorrentino, Rucco, Baselice *et al*., 2021a; Sorrentino, Seguin, Rucco *et al*., 2021b). Once recognized, the components were removed, and the sensor signal reconstructed. Finally, since the elimination of noisy recording segments cut the recordings in several trials, we only considered trials longer than 4 s, in order to guarantee the convergence of the connectivity estimates (Fraschini *et al*., 2016) (Fig. 1b,c).

### Source reconstruction

The data underwent a beamforming procedure using the FieldTrip toolbox. For each participant, the fiducial points were visually recognized on the native MRI and used to co‐register the MEG acquisition. We used Nolte's (2003) conduction model and implemented the linearly constrained minimum variance beamformer (van Veen, van Drongelen, Yuchtman & Suzuki, 1997) to reconstruct time series related to the centroids of 116 regions of interest (ROIs), based on the Automated Anatomical Labeling atlas (Gong, He, Concha *et al*., 2009; Hillebrand, Tewarie, van Dellen *et al*., 2016). Because of the position of the MEG sensors, signals coming from the cerebellum were excluded as unreliable and we considered only the first 90 ROIs (Fig. 1d).

### Construction of brain networks

To estimate the connectivity among the 90 ROIs, we applied the Phase Linearity Measurement (PLM) index (Sorrentino, Ambrosanio, Rucco & Baselice, 2019), an adirectional measure based on the phases of signals, defined by the following equation (Baselice, Sorriso, Rucco, & Sorrentino, 2019):

$$\mathrm{PLM}=\frac{\int_{-B}^{B}¦\int_{0}^{T}e^{i\Delta \emptyset \left(t\right)}e^{-i2\pi \;f\;t}\mathrm{dt}¦2\;\mathrm{df}\;}{\int_{-\infty }^{\infty }¦\int_{0}^{T}e^{i\Delta \emptyset \left(t\right)}e^{-i2\pi \;f\;t}\mathrm{dt}¦2\;\mathrm{df}\;},$$

where ∆∅(*t*) is the phase difference between two signals, –B and B is the bandwidth, *f* is the frequency and *T* is the observation period.

*PLM* was computed for each pair of brain regions, obtaining a 90 × 90 weighted adjacency matrix for each trial of registration. The five canonical frequency bands were estimated as delta (0.5–4 Hz), theta (4.0–8.0 Hz), alpha (8.0–13.0 Hz), beta (13.0–30.0 Hz) and gamma (30.0–48.0 Hz). Thereafter, we built a brain network starting from each adjacency matrix (Stam, Nolte & Daffertshofer, 2007), where each of the 90 brain regions were represented as a node, and the weighted edges between two nodes were represented by PLM values. Then, applying Kruskal's algorithm (Kruskal, 1956) to each reconstructed brain network, we calculated the minimum spanning tree (MST), a loopless graph with N nodes and M = N‐1 links. In this way, we obtained topologic measures unaffected by the degree distribution, matrix density, or arbitrary thresholds (van Wijk, Stam, & Daffertshofer, 2010) (Fig. 1 e,f). For generalization purpose, the analyses have been repeated using different metrics. The connectivity has been estimated using the Phase Locking Value (PLV) (Lachaux, Rodriguez, Martinerie & Varela, 1999), while the network characteristics have been analyzed using both orthogonal minimum spanning tree (OMST) (Dimitriadis, Antonakakis, Simos, Fletcher & Papanicolaou 2017; Dimitriadis, Salis, Tarnanas & Linden, 2017) and efficiency cost optimization (ECO) (De Vico Fallani, Latora & Chavez, 2017) methods. For a detailed explanation please refer to Data S1.

### Graph theoretical analysis

We calculated both global and nodal (regional) parameters. To obtain indices representative of the global topological organization of brain networks, we calculated the following: the *leaf fraction* (Boersma, Smit, Boomsma *et al*., 2013), which is the fraction of nodes with a degree of 1, an expression of the network integration; the *degree divergence*, a measure of the broadness of the degree distribution, related to the synchronizability of the networks (Stam, Tewarie, Van Dellen, van Straaten, Hillebrand & Van Mieghem, 2014; Tewarie, van Dellen, Hillebrand & Stam, 2015); the *tree hierarchy* defined as the trade‐off between network integration and central nodes overload; the *diameter*, defined as the longest shortest path of an MST; and the *assortativity*, an estimation of the relationship among nodes.

To examine the regional features of the network we calculated two nodal parameters: the *degree*, defined as the number of connection incidents on a given node, and the *betweenness centrality* (BC), described as the number of shortest paths passing through a given node over the total number of shortest paths in the network (Tewarie *et al*., 2015). To obtain a single value of each parameter for each participant, the metrics were averaged across the trials of each subject.

### MRI acquisition

MRI images of seventy participants were acquired using a 1.5‐T Signa Explorer scanner with an 8‐channel parallel head coil (General Electric Healthcare, Milwaukee, WI, USA). We obtained three‐dimensional T1‐weighted images (gradient‐echo sequence Inversion Recovery prepared Fast Spoiled Gradient Recalled‐echo, time repetition = 8.216 ms, TI = 450 ms, TE = 3.08 ms, flip angle = 12, voxel size = 1 × 1 × 1.2 mm1; matrix = 256 × 256). We used a standard template MRI for seven participants who refused to undergo the MRI examination.

### Statistical analysis

Analyses were performed using MATLAB (MathWorks, version R2013a, Natick, MA, USA). Both global and regional metrics were correlated, using Pearson's correlation, to the seven TCI scores in each frequency band. Further analyses comparing male and female individuals were performed through a permutation test, in which each subject label has been permuted 10,000 times. A significance level of 0.05 was applied after false discovery rate (FDR) correction across 90 regions and temperaments/characters.

## RESULTS

### Topological brain network parameters

**Fig. 2:**
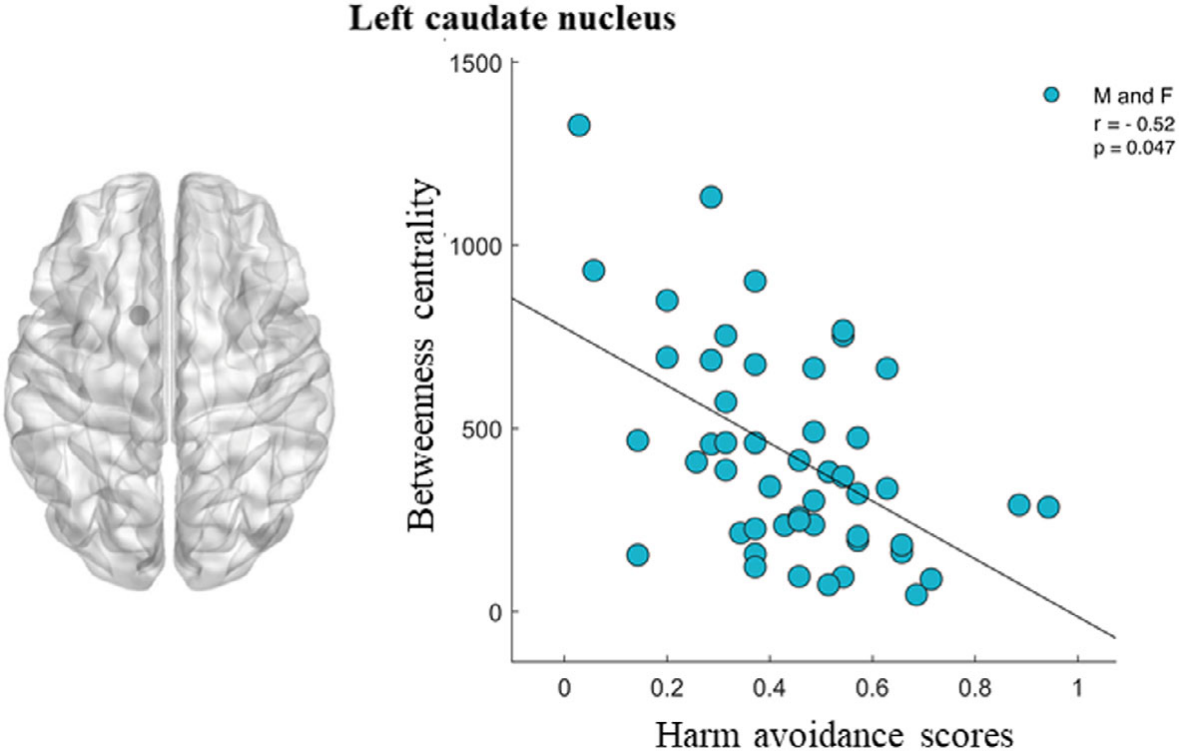
Correlation in the alpha band between the betweenness centrality of the left caudate nucleus and the harm avoidance trait. In the left part a reconstruction of the caudate nucleus is shown. [Colour figure can be viewed at wileyonlinelibrary.com]

A Pearson's correlation analysis was performed to evaluate the relationship between both global and nodal topological parameters and seven dimensions of personality (four temperaments and three characters), according to TCI, in 50 male and female participants. No global topological parameter showed correlation with any of the TCI dimensions. On the contrary, in relation to nodal parameters, we found a significant negative correlation in the alpha band involving the betweenness centrality (BC) of the left caudate (LC) and the harm avoidance (HA) trait (*r* = −0.52; *p* = 0.047) (Fig. 2).

### Comparison between male and female gender

**Fig. 3:**
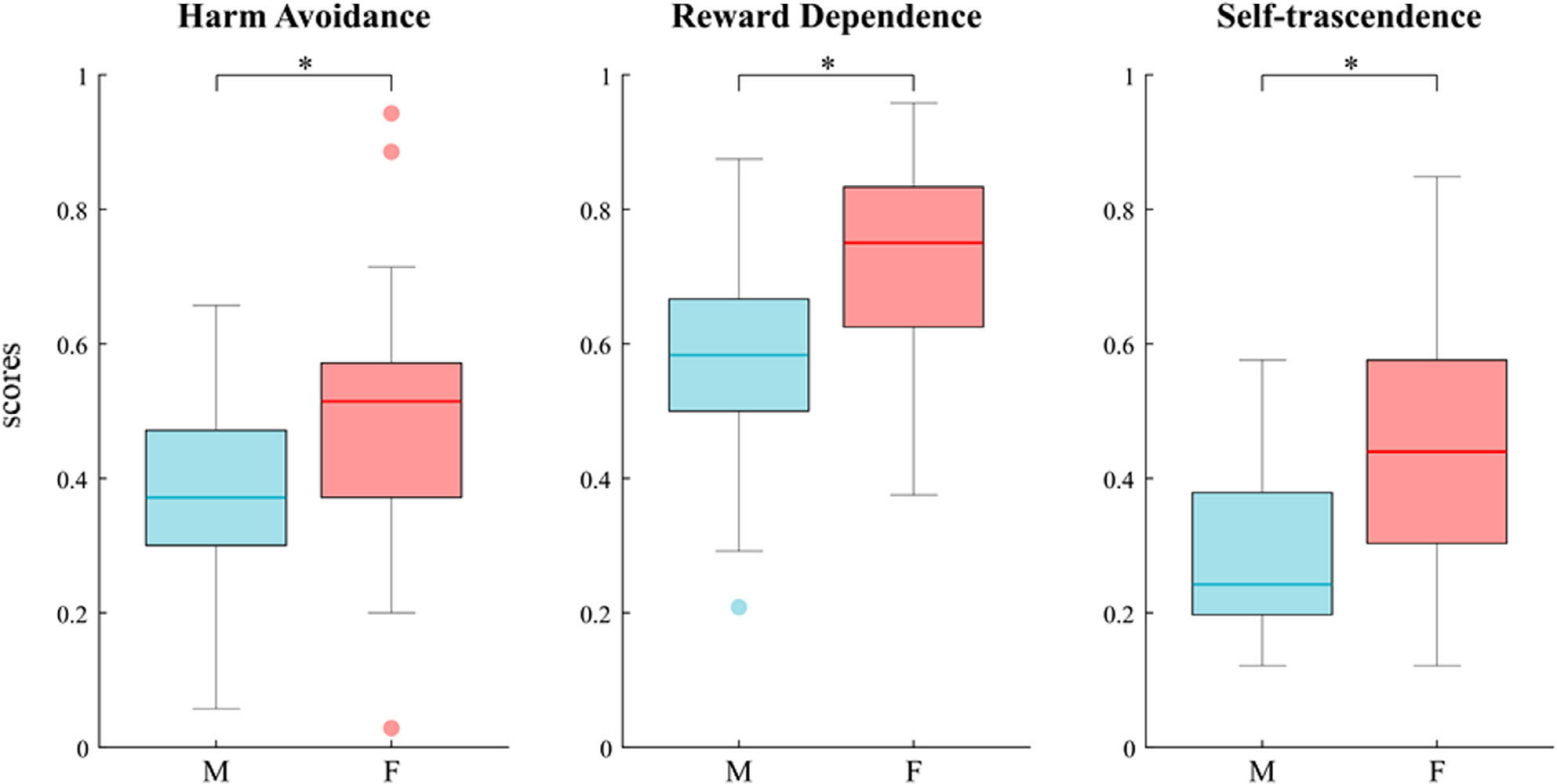
Comparison between males and females in TCI. The asterisks indicate statistical significance *p* < 0.05. [Colour figure can be viewed at wileyonlinelibrary.com]

Comparing male and female population in TCI by means of permutation analysis, we found that women scored higher than men in HA (*p* = 0.048) and RD (*p* = 0.041) temperaments and ST (*p* = 0.027) character (Fig. 3).

**Fig. 4:**
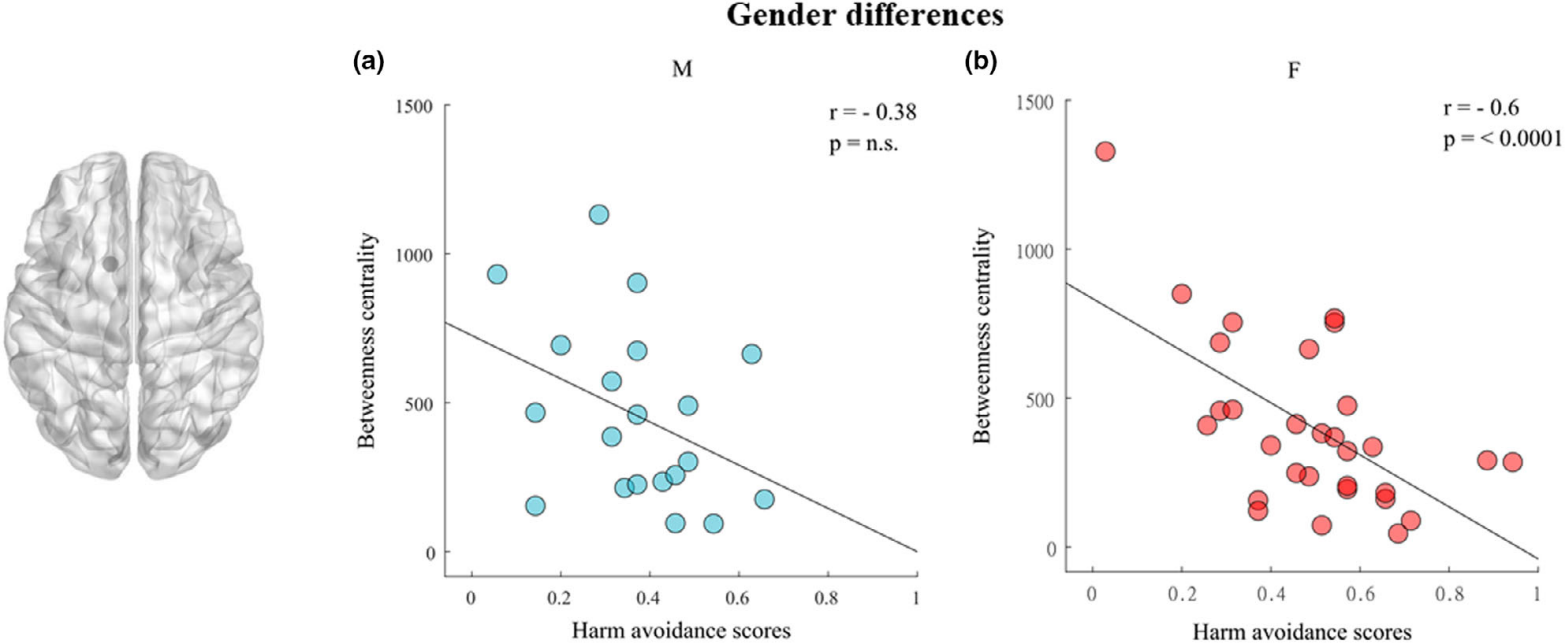
Correlation in the alpha band between the betweenness centrality of the left caudate nucleus and the harm avoidance trait in relation to males (a) and females (b). [Colour figure can be viewed at wileyonlinelibrary.com]

Having detected a significant negative correlation in alpha band between left caudate and HA when the entire sample was taken into consideration, we examined in detail this correlation considering each gender separately. While in male individuals, the correlation did not show to be significant (*r* = −0.38; *p* = 0.098) (Fig. 4a), in female participants it was significant, with a negative coefficient (*r* = −0.6; *p* < 0.001) (Fig. 4b) showing thus the same pattern as when the two groups are processed together.

The analyses repeated using different metrics (i.e., PLV, OMST, ECO) did not show any significant result. For a detailed description please see Data S1.

## DISCUSSION

In this study, we used MEG to investigate the brain topology correlated with personality traits in healthy individuals. Assuming the brain as a coherent interconnected network, we analyzed how individual differences in Cloninger's temperaments and characters correlated with specific cerebral structures synchronized among themselves. We found high HA scores were associated with a reduced centrality of the left caudate nucleus (Fig. 2). In interpreting this data, it can be useful to recall the meaning of this temperament and the functional role of the caudate nucleus. HA consists of the tendency to inhibit behaviors, to act with caution and apprehension. The adaptive advantages of high HA scores are cautiousness and careful planning when a hazard is likely to happen, while the disadvantages occur when a hazard is unlikely to happen, but its anticipation leads to maladaptive inhibition and anxiety (Laricchiuta, Petrosi *et al*., 2014). For these characteristics, HA is the trait that is most correlated to the salience of the environmental stimuli and reflects some features of the caudate nucleus; it plays a key role in adaptive behavioral responses and in choosing actions that are likely to lead to a positive outcome (Tricomi, Delgado, & Fiez, 2004). Notably, the caudate nucleus, as part of the striatum, is involved in the motivation (Ernst & Fudge, 2009) and anticipation of a potential reward (Benningfield, Blackford, Ellsworth *et al*., 2014). In addition, its activation has been linked to attentional bias toward positive social stimuli (Dedovic, Giebl, Duchesne *et al*., 2016; Haruno, Kuroda, Doya *et al*., 2004; Pizzagalli, Holmes, Dillon *et al*., 2009).

The association between HA and the left caudate nucleus is specific only for the BC nodal parameter, which denotes the shortest path between nodes in the interconnected whole‐brain network and in turn the importance of the left caudate nucleus in mediating communication between the entire network of nodes (Freeman, 1977). In other words, the BC parameter suggests that the more behaviors are inhibited (high HA values), the less the caudate nucleus is a central node in the network. Similarly, we can suggest that the more the behaviors are goal directed (low HA values), the more the caudate nucleus become a central node in the network. Thus, the reduced centrality of the caudate nucleus associated with HA scores resonates well with the assumption that individuals scoring higher in this temperament will report greater behavioral inhibition and greater tendency to process stimuli as potentially threatening rather than potentially rewarding (Cloninger *et al*., 1994). In addition, higher HA scores have been found to be predictive of anxiety disorders, and several studies have documented that anxiety disorders are characterized by alterations in caudate structure and connectivity and altered stimulus processing (Sareen, Campbell, Leslie *et al*., 2007; Whiteside, Port, Deacon, & Abramowitz, 2006). Finally, the caudate nucleus' reduced centrality associated with higher HA scores is in accordance with previous fMRI studies reporting a relationship between HA scores and connectivity in the cortico‐limbic circuit (Westlye, Bjørnebekk, Grydeland, Fjell & Walhovd, 2011), in which the caudate nucleus plays a key role also in adaptive emotional responses. Of note, using resting‐state functional connectivity data, HA has been found to be negatively related to the insular salience network's efficiency (Markett, Montag, Melchers, Weber & Reuter, 2016), thus suggesting a crucial role for the caudate nucleus in this large‐scale network.

In the present study, we also analyzed the gender differences in the TCI scores and correlated them to specific topological data. We found that women scored higher than men in HA and RD temperaments and ST character (Fig. 3), confirming previous well documented findings (Costa, Terracciano & Crae, 2001; Schmitt, Realo, Voracek & Allik, 2008).

The negative correlation we found between the left caudate and HA for the BC nodal parameter, induced us to analyze it by considering each gender separately. Only female participants showed the same pattern when the two groups were processed together. In fact, this correlation was maintained in the female subgroup and collapsed in the male one (Fig. 4a,b). This difference can be explained by dissimilarities in personality traits (Chapman, Duberstein, Sörensen & Lyness, 2007; Costa *et al*., 2001; Weisberg, Deyoung & Hirsh, 2011) as well as in brain structural and functional asymmetries between female and male groups (Wang, Zhan, Yan *et al*., 2019). Our topological data can also be discussed in relation to a previous voxel‐based morphometry study that investigated the association between gray matter volume (GMV) and personality traits in men and women using the NEO five‐factor inventory (NEO‐FFI). Among various results, the significant association between GMV and neuroticism that was found only in men, suggests that brain structure–personality relationships are highly dependent on gender (Nostro *et al*., 2017). Although the NEO‐FFI is different from the TCI, there are many similarities between HA and neuroticism. In fact, neuroticism is associated with a decreased ability to ignore irrelevant information (Prabhakaran *et al*., 2011). It has also been related to a “hypervigilance of threats” (Mogg & Bradley, 1998; Richards *et al*., 2014); that is, an adaptive behavior to perceive a potential risk faster, which comes at the cost of specificity and, consequently, less successful inhibition of irrelevant stimuli and response sets. Like us, Nostro *et al*. (2017) found neuroticism scores were higher in women than men. Unlike us, they found structural brain asymmetries only in the male group, suggesting that, in the female group, brains may be more decentralized (Zaidi, 2010) and may feature stronger interhemispheric structural connectivity (Ingalhalikar, Smith, Parker *et al*., 2014), a factor that may benefit multitasking characteristics (Zaidi, 2010). Given Nostro and colleagues' interpretation, we can hypothesize that the characteristic of multitasking is reflected in the organization of the large‐scale networks that include multiple brain regions. Thus, in this intriguing hypothesis, the insular salience network would be wider and would include the caudate nucleus.

Beyond the interpretation of our data, other connectivity studies showed that men had higher nodal efficiency of the internal regions such as insula and peripheral lingual gyrus, while women showed significantly greater betweenness centrality in the precuneus and peripheral superior occipital gyrus (Wang *et al*., 2019; Yan, Gong, Wang *et al*., 2011), thus evidencing a clear difference between women and men in large scale brain network organization.

This study has some limitations that naturally suggest using caution in generalizing these findings. The first limitation is the exclusion of the cerebellum from the analysis of the networks. This exclusion is a consequence of our MEG system which does not cover the lower occipital area and therefore does not allow a clean recording of the signal coming from the cerebellar nuclei. It is important to recognize this limitation in light of recent evidence suggesting that the cerebellum together with the basal ganglia influence and sustain processes that are linked to individual differences in approach and avoidance behaviors through a cortico‐basal‐cerebellar loop (Laricchiuta, Petrosini, Piras *et al*., 2014; Picerni *et al*., 2013). Furthermore, the analyses repeated using different synchronization and network filtering measures could not confirm the results (please see Data S1). It should be noted that a different filtering approach may highlight different topological brain features, and caution should be observed in data interpretation. However, the PLM showed to be a very reliable measure. Indeed, in a previous study it was demonstrated that PLM, compared to other metrics such as Phase Lag Index and Amplitude Envelope Correlation, displayed greater robustness to noise, and insensitivity to volume conduction. Moreover, the PLM requires significantly less data in order to provide reliable results, with respect to the abovementioned measures (Baselice *et al*., 2019).

In addition, the relatively small sample prohibits drawing conclusion about gender differences in brain underpinnings of personality mainly for females, more affected by hormonal fluctuation (Liparoti, Troisi Lopez, Sarno *et al*., 2021). However, the differences between the groups in our study encourage us to continue this line of research to better understand the topological organization in both. This study also has several strengths. First, it is one of the first studies that uses MEG to investigate the biological substrates of personality traits and as explained in the introduction, this analysis system allows us to obtain precise and accurate results. Second, although small, our sample consists of young adults of similar age. This feature represents a great advantage because the human brain changes during one's life span and analyzing the brains of individuals of the same age allows us to gain an accurate picture of brain topology at that age. Finally, while beyond the scope of the current work, it should be kept in mind that considering multiple network variables (as well as the same feature across multiple frequencies) might further improve the correlation. However, given the fact that the network features that are estimated in this work vary non‐linearly, using a multilinear model is not guaranteed to converge. Hence, we have opted to keep the analysis of the correlations between network features and personality traits bivariate. However, this should be kept in mind when interpreting our results.

## CONCLUSION

In conclusion, our results highlight that the topological measures in MEG studies offer unique information about the neurobiological basis of personality traits. These results also pave the way to exploring the mutual influences of topological and functional connectivity in neural communication efficiency and disruption as biomarkers of psychopathological personality traits.

This research was supported by funding from the Department of Humanities, University of Naples “Federico II” (Fondi ricerca dipartimentale 30% and 70%, 2020/2021) to L.M. Funding provided by Universita degli Studi di Napoli Federico II within the CRUI‐CARE Agreement. This research did not receive any specific grant from funding agencies in the public, commercial, or not‐for‐profit sectors.

## CONFLICT OF INTEREST

The authors declared no potential conflicts of interest with respect to the research, authorship, and/or publication of this article.

All authors designed the research. Emahnuel Troisi Lopez, Valentina Colonnello, and Marianna Liparoti performed the research. Emahnuel Troisi Lopez, Valentina Colonnello, Mauro Castaldi, Fabio Alivernini and Pierpaolo Sorrentino analyzed the data. Emahnuel Troisi Lopez, Valentina Colonnello, Fabio Alivernini, Paolo Maria Russo, Giuseppe Sorrrentino, Fabio Lucidi, Laura Mandolesi and Pierpaolo Sorrentino wrote the paper. All authors read, revised, and approved the final manuscript.

## Supporting information


**Data S1.** Supplementary Material

## Data Availability

The data that support the findings of this study are available from the corresponding author upon request.
