# Supplementary material for: Brain network topology and personality traits: A source level magnetoencephalographic study
Source: Scand J Psychol. 2022 Jun 8;63(5):495–503. doi: 10.1111/sjop.12835 (PMC9796445; doi:10.1111/sjop.12835)
Supplement: Supplementary file 1 — Data S1. Supplementary Material [file SJOP-63-495-s001.docx]

Manuscript title: Brain network topology and personality traits: a source level magnetoencephalographic study

Here we describe the supplementary analyses, performed using different metrics, both for the connectivity and the network estimation.

Alternatively to the Phase Linearity Measurement (PLM) (Sorrentino et al., 2019), we used the Phase Locking Value (PLV) (Lachaux et al., 1999) for the connectivity estimation. This measure is built assuming that two functionally connected brain regions would show a quite constant difference between the instantaneous phases of their signals.

For the network estimation, we used two different methods. The first is called orthogonal minimum spanning tree (OMST) (Dimitriadis et al., 2017a, 2017b), a method attempting to individuate the optimal communication within the network through the global efficiency – cost formula, choosing the connections through orthogonal minimum spanning tree networks. The latter is named efficiency cost optimization (ECO) (De Vico Fallani et al., 2017), and approach creates a density threshold that eliminate the weakest edges and optimize the trade-off between the network efficiency and the cost of its connections.

We repeated the correlation analysis using the abovementioned measures. However, we could not find any significant correlation between topological measures and personality scores. This different finding might be due to the higher noise-resiliency of the PLM (Baselice et al., 2019), or to different rejection properties with respect to field-spread. Moreover, the different filtering methods may have highlighted different topological characteristics.

REFERENCES

Baselice, F., Sorriso, A., Rucco, R., & Sorrentino, P. (2019). Phase linearity measurement: a novel index for brain functional connectivity. *IEEE transactions on medical imaging*, 38, 873-882. https://doi.org/10.1109/TMI.2018.2873423

De Vico Fallani, F., Latora, V., & Chavez, M. (2017). A topological criterion for filtering information in complex brain networks. *PLoS computational biology*, 13(1), e1005305.

Dimitriadis, S. I., Antonakakis, M., Simos, P., Fletcher, J. M., & Papanicolaou, A. C. (2017a). Data-driven topological filtering based on orthogonal minimal spanning trees: application to multigroup magnetoencephalography resting-state connectivity. *Brain connectivity*, 7(10), 661-670.

Dimitriadis, S. I., Salis, C., Tarnanas, I., & Linden, D. E. (2017b). Topological filtering of dynamic functional brain networks unfolds informative chronnectomics: a novel data-driven thresholding scheme based on orthogonal minimal spanning trees (OMSTs). *Frontiers in neuroinformatics*, 11, 28.

Lachaux, J. P., Rodriguez, E., Martinerie, J., & Varela, F. J. (1999). Measuring phase synchrony in brain signals. *Human brain mapping*, 8(4), 194-208. https://doi.org/10.1002/(SICI)1097-0193(1999)8:4%3C194::AID-HBM4%3E3.0.CO;2-C

Sorrentino, P., Ambrosanio, M., Rucco, R., & Baselice, F. (2019). An extension of Phase Linearity Measurement for revealing cross frequency coupling among brain areas. *Journal of NeuroEngineering and Rehabilitation*, 16, 135. https://doi.org/10.1186/s12984-019-0615-8
